# A systematic scorecard-based approach to site assessment in preparation for Lassa fever vaccine clinical trials in affected countries

**DOI:** 10.1186/s40814-020-00567-4

**Published:** 2020-02-13

**Authors:** Kolawole Salami, Nathalie Imbault, Aljoscha Erlebach, Johanna Urban, Mike Zoglowek, Nadia G. Tornieporth

**Affiliations:** 1 World Health Organization, Geneva, Switzerland; 2 Coalition for Epidemic Preparedness and Innovations, London, UK; 3 Department of Information and Communication, Hochschule Hannover - University of Applied Sciences and Arts, Faculty III, Hannover, Germany

**Keywords:** Lassa fever vaccines, Clinical trials, Endemic countries, Assessment, Site selection, Scorecard, Site assessment, Capacity strengthening

## Abstract

**Background:**

We sought to develop and test an objective scorecard-based system for assessing and categorizing available research sites in Lassa fever-affected countries based on their preparedness and capability to host Lassa fever vaccine clinical trials.

**Methods:**

We mapped available clinical research sites through interrogation of online clinical trial registries and relevant disease-based consortia. A structured online questionnaire was used to assess the capability of clinical trial sites to conduct Lassa fever vaccine clinical trials. We developed a new scoring template by allocating scores to questionnaire parameters based on perceived importance to the conduct of clinical trials as described in the WHO/TDR Global Competency Framework for Clinical Research. Cutoff points of 75% and 50% were used to categorize sites into categories A, B, or C.

**Results:**

This study identified 44 clinical trial sites in 8 Lassa fever-affected countries. Out of these, 35 sites were characterized based on their capacity to hold Lassa fever vaccine clinical trials. A total of 14 sites in 4 countries were identified as ready to host Lassa fever vaccine trials immediately or with little support.

**Conclusion:**

It is feasible to hold Lassa fever vaccine trials in affected countries based on the outcome of the survey. However, the findings are to be validated through sites’ visits. This experience with a standardized and objective method of the site assessment is encouraging, and the site selection method used can serve as an orientation to sponsors and researchers planning clinical trials in the region.

## Introduction

Immunization is one of the most beneficial and cost-effective disease prevention measures. There are global efforts to develop new vaccines for the control of diseases of global health importance. Africa has not featured prominently on the world’s vaccine clinical trial landscape [1] despite being the second most populated continent, and alongside Asia, the hub of most infectious diseases [2, 3]. As of September 24, 2018, a snapshot of total registered vaccine clinical trials on the US National Institute of Health (NIH) Library of Medicine’s portal revealed 6677 ongoing vaccine clinical trials across the world with only 347 (~ 5%) taking place in Africa. However, there is a disproportional distribution of vaccine trials on the African continent with approximately a third of these vaccine trials attributable to the Republic of South Africa (RSA). West Africa, including all Lassa fever (LF)-affected countries, contributed 35% (121) of Africa’s vaccine trials; however, most of these trials (~ 83%) occurred in Mali, Burkina Faso, Guinea-Bissau, Gambia, and Senegal. Out of the 8 Lassa-affected countries, the three worst-hit (Nigeria, Sierra Leone, and Liberia) account for only 12% of vaccine trials in West Africa, further underlining the gap and inequity in the distribution of this critical component of Research and Development (R&D) [4]. It should also be noted that only 22% of these studies were phase I trials with the majority being phase II, III, and post-vaccine introduction effectiveness studies [4].

Clinical research demands careful consideration of ethical, scientific, methodological, and operational aspects of each study [5]. Many factors play a role in the appropriateness of site selection for sponsored multi-center clinical studies. These include having access to patients meeting the eligibility criteria, expertise in the outcome (disease) of interest, presence of facilities or equipment, and trained investigators willing and able to perform the research. Also, previous experience with clinical trials, especially involving vaccines, is a useful indicator of successful clinical trial management. Other factors include having the required equipment, personnel, and procedures to handle the investigational product (IP). Familiarity with trial procedures, e.g., contracting, informed consent process, and submissions to ethical and regulatory authorities, is also an important consideration. Of recent, a significant number of clinical trials are experiencing delays due to many factors, the majority of which are associated with the appropriateness of the clinical trial site and challenges with patient recruitment [5–7]. The inability to recruit enough study subjects increases the length of the enrollment period with the attendant increase in operational cost. The time and cost of maintaining a site between and during clinical trials could gulp a considerable amount of funds, which under-performing sites would not be able to justify. Also, sites with inexperienced or unskilled investigators are more likely to incur protocol infractions or to have low-quality data that will require further training, on-site visits, and more queries for clarification, all of which impacts costs and study duration [7].

Although North America and Western Europe continue to lead the world’s clinical trial map, patients’ recruitment remains a challenge, causing significant delays (plus additional cost) to the conduct of clinical trials in these countries. A reduction in time and cost of conducting a clinical trial has become more critical as competition to bring new drugs and vaccines to the market is increasing. These challenges have prompted most sponsors to look towards conducting clinical trials in developing countries of Asia, Latin America, Central and Eastern Europe, the Middle East, and Africa [6]. These regions are under-represented in research, yet these populations stand to gain more from research as their infectious diseases’ burden and the need for effective vaccines is higher than that of developed countries. Conducting clinical trials in Africa is deemed challenging because of perceived problems with getting valid informed consent, ethical compensation mechanisms for indigent populations, poor health infrastructure, and considerable socio-economic and cultural divides. However, these countries could also offer an attractive setting for clinical trials [8]. Conducting clinical trials in sub-Sahara Africa promotes access to innovative medical countermeasures and enhances the research capacities of scientists in these countries [9]. Importantly, clinical trials need to be responsive to the needs of host communities. Based on the high prevalence of Lassa fever in West Africa, it is important to conduct the clinical trials within the setting to allow for an assessment of the potential impact of factors such as disease epidemiology, health service needs, social determinants of health, presence of comorbidities, compliance, and genetic make-up on the efficacy and effectiveness of the eventual vaccine [10]. This also ensures that recommendations of the use of Lassa virus (LASV) vaccine in these countries are based on appropriate benefit-risk assessments especially because available evidence suggests that differences might exist in the rate of enzyme-mediated metabolism of drugs and biologics between different populations based on genetic mutations [11].

However, there are significant challenges in the clinical testing of LASV vaccine candidates and the eventual delivery to populations in need. Conducting clinical trials requires the presence of experienced researchers and sites capable of carrying out high-level, good clinical practice (GCP) compliant scientific trials. Site selection should also be supported by current epidemiological data on the target disease. A lot of the required information remains unknown for the LF-affected countries. Choosing the right site can save time, money, and ultimately lives. Therefore, this study is aimed at determining the presence and preparedness of research sites in LF-affected countries to host a successful LF vaccine clinical trial. In this study, we reviewed the literature to determine the vaccine clinical trial landscape in West Africa, mapped the potential clinical research sites in LF-affected countries, and developed a scorecard for the assessment and operational characterization of the mapped sites. The findings from this study could serve as a guide to vaccine developers who may lack experience in conducting clinical trials in West Africa. It can also serve as a useful resource for organizations committed to developing research capacity in the sub-region.

## Method

### Site mapping

We mapped potential trial sites in LF-affected countries by interrogating clinical trial registries to identify sites that have conducted trials in the past 6 years [6] (January 2012–June 2018). For this study, a LF endemic country was defined as any country that has reported a human case of LF from an autochthonous transmission; these include Nigeria, Ghana, Benin, Sierra Leone, Liberia, Guinea, Togo, Mali, and Burkina Faso. Clinical trial registries surveyed include www.clinicaltrials.gov. international clinical trials registry platforms, e.g., WHO/ICTRP, the pan-African clinical trial partnership (http://www.pactr.org/), and the global health network site finder (http://sitefinder.tghn.org/). Also, sites active in conducting clinical research on viral hemorrhagic fevers in affected countries as listed on http://vhfc.org/consortium/ and http://www.edctp.org/ were included even if no significant clinical trials were conducted over the earlier defined period.

### Site assessment

Assessment of the mapped sites was done through a focused survey utilizing a questionnaire containing open- and closed-ended questions aimed at determining the level of facilities’ preparedness to conduct LASV vaccine trials. The questionnaire was an adapted version of a pretested and validated site readiness checklist for vaccine trials obtained from the global health training network web resources [12]. The checklist template was edited to include questions pertinent to the conduct of LASV vaccine clinical trials. These inquiries include physical accessibility of the research site, presence of infrastructures for LF diagnosis, site’s experience with conducting clinical trials, research output or publications, and access to facilities for the storage of biological samples. Also, a specific sub-section on X-rays was removed in favor of questions aimed at determining the overall clinical care ability of sites. The adapted questionnaire was pretested by sending to 12 research sites in West Africa with 3 sites (1 each) from Senegal, Côte d’Ivoire, and Niger responding. The only adjustment made after the pilot was to increase the space for listing research outputs or publications emanating from research activities carried out at the sites. The questionnaires were sent out as emails with an active hyperlink to the mapped sites in LF-affected countries. The questionnaire was also shared on social media outlets like Twitter and LinkedIn to ensure a wider audience was reached. This was also to provide opportunities for sites that might have been missed in the mapping exercise to respond. Each research site was given 3 weeks to respond to the questionnaire.

### Data analysis

A scorecard was developed to assess the clinical trial sites and categorize them based on predetermined criteria (Additional file 1: Table S1). The questions sought information on four broad categories based on the WHO/TDR competency wheel [13]: these include scientific thinking, ethics, quality/risk management, study and site(s) management, and research operations. A quantitative score was allocated to each question in an empirical, but systematic fashion based on the lead author’s experience and perceived importance of the question to the conduct of LF vaccine clinical trials. The score was calculated in Excel using “if-conditions” in the formulas, e.g., if the question “Is the site accessible by road?” was answered with “Yes,” the score column was automatically set to 1, any other answer led to “No.” The more answers that were possible, the more nesting for the if-conditions that were necessary. For the others, multiplying or dividing factors were used as appropriate, e. g., a previous history of successful conduct of vaccine clinical trial carried twice the score of a previous drug clinical trial. The total number of staff per site was divided by a factor of 2 (up to 32 staff) to give a maximum score of 16. Also, having the ability to conduct nucleic acid-based diagnosis (PCR) attracted a score of 10 due to its importance to LF diagnosis. Analysis of the quantitative data and calculation of the scores per site were done using a Microsoft Excel-based template aimed at categorizing the trial sites based on their score over a defined total maximum score of 100. Prior to receiving the responses from the sites, it was predetermined that sites with scores ≥ 75% would be categorized as “A” sites, 50–74% as “B” sites, and < 50% as “C” sites, while non-responding sites or sites that declined participation will be categorized as “D” sites. The 75% threshold was based on the minimum score possible for any site with facilities, personnel, and processes deemed necessary by the authors for the conduct of LASV vaccine clinical trials as displayed in Table 1.

**Table 1:** Minimum facilities required to hold a Lassa fever vaccine trial

											Score
Scientific thinking	Design and planning of research	Protocol optimization	Interpretation of study results	What is the total number of scientific staff at the clinical research center?							10
				How many years has this site been in existence?							4
				List 5 more recent publications emanating from research conducted at the site							5
				List top 5 pharmaceutical companies or global health funding agencies you have worked with							5
				**Total**							**24**
Study and site management	Study oversight	Human resources	Resources management	What is the total number of non-scientific staff at the clinical research center?							10
				Distance from airport (domestic/international) in kilometers							2
				Is the site accessible by road?							1
				What is the road condition (e.g., dirt, tarmac)?							1
				Administrative offices							1
				Access to reliable internet connection? State type (wifi/DSL/dongle/cable/satellite)							1
				Designated study document storage area and archiving facilities available on site (e.g., archiving room)							1
				Power source with dedicated functional back up available?							1
				Vaccine management (reception, local shipment), cold chain management, destruction, vaccination process, and equipment							1
				Dedicated + 2 and + 8° refrigerators? If yes, indicate how many in spaces below							1
				Access to − 20 °C freezers? If yes, indicate how many in space below							1
				Access to − 70 °C freezers? If yes, indicate how many in space below							1
				**Total**							**22**
Research operations	Data management	Clinical and laboratory	Interaction with community and participants	Outpatient care facility							1
				Inpatient care facility for study patients							1
				Examination room(s) for subject evaluation and treatment							1
				Access to laboratory services?							1
				If no laboratories on site, what is the distance between the study/trial site and the laboratory(ies)? (Please indicate miles or kilometers)							2
				List below the different laboratories available such as hematology, clinical chemistry, etc.							6
				Is the laboratory able to conduct immunochemical studies, e.g., RT-PCR?							10
				Does the laboratory have local/national reference ranges?							1
				Dedicated work area for clinical research team							1
				Consenting or counseling room(s)							1
				Presence of or easy access to intensive care unit?							1
				How many kilometers away is the ICU (if not on site)?							1
				Emergency services (e.g., where anaphylaxis can be handled)?							1
Ethics, quality, and risk management	Regulations and governance			Is there an established quality management system in place?							1
				**Total**							**29**
										**Total**	**75**

## Results

A total of 44 sites were identified in 8 LF-affected countries (Nigeria, Ghana, Sierra Leone, Liberia, Benin, Mali, Burkina Faso, and Guinea) through clinical trial registries and viral hemorrhagic fever consortia. No site was identified in Togo. Additionally, 5 sites (2 from Senegal, 2 from Cote D’Ivoire, and 1 from Niger) responded to the online call but were excluded from the analysis based on the scope of this study. At the end of week 1, only 7 (16%) out of the mapped 44 sites had responded. This number increased to 13 (30%) at the expiration of the 3-week deadline. Phone calls, email reminders, and networking through professional colleagues and contacts in these countries were intensified over the next 3 weeks. The period of data collection was extended by a further 3 weeks to allow all sites an opportunity to respond. Finally, the database was closed at the end of week 9, with 35 (79.5%) sites giving a favorable response and 2 (4.5%) sites declining to participate citing lack of interest in conducting clinical trials at this time, while no response was obtained from 7 sites (16%).

### Descriptive analysis

A total of 14 sites (32%) were categorized as category A sites, 18 (41%) as category B, 3 (7%) as category C, and 9 (20%) sites in category D. Most of the research institutions were government-owned institutions. The average period of sites’ existence was 26.5 years (SD 20). However, 9 (26%) of the 35 sites were established within the last 7 years. Overall, 6 (17%) of the 35 surveyed sites had no drug or vaccine clinical trial experience. All sites were accessible by road, tarmac roads in all except 4 (11%) of the 35 sites. The average distance to the nearest airport was 110 km (SD 231). An average of 12 clinical trials had been conducted at these sites since inception (SD 14), with only an average of 3 being vaccine trials (SD 4.9) and 6 drug trials (SD 10.7). These weighted averages were, however, strongly influenced by drugs and vaccine trial experiences of sites in Mali, Burkina Faso, and Ghana.

All surveyed sites (except for category C sites) appeared to have adequate scientific and support staff with an average of 33 (SD 33.5) scientific staff and many more support staff per site. All sites had experience with community mobilization and engagement with civil society organizations (CSO). Overall, 30 (85%) of the 35 sites had quality management systems in place. While 32 of the 35 sites (91%) had access to patients for recruitment through outpatient clinics or community engagement facilities, 12 (34%) sites had no inpatient facilities. An immediate access to emergency medical care was found only in the 17 (48.5%) sites situated within teaching hospitals. However, most sites had partnership arrangements and/or a memorandum of understanding (MOU) with nearby hospitals to provide emergency clinical care to study subjects enrolled in clinical trials. Five sites (14%), however, did not have any access to an intensive care unit (ICU).

Overall, 31 (89%) out of the 35 sites had access to − 20 °C, − 70 °C, + 2 °C, and + 8 °C refrigerators. In addition, 7 (20%) sites confirmed the availability of − 80 °C freezers, creating an opportunity for long-term biological samples storage. All the 35 sites had dedicated staff for data entry, and there was a possibility and willingness to upgrade the number of data management staff based on available funding and scope of planned clinical trial. Overall, 29 (83%) sites had archives for storing study documents.

## Discussion

Lassa fever has remained a significant cause of morbidity and mortality in the West African sub-region since its identification in 1969 [14]. Nigeria, Sierra Leone, and Liberia have borne the major burden of the disease. In Nigeria, the disease afflicted more than 1000 and killed nearly 300 between the year 2018 and 2019 [15]. There are currently no vaccines or satisfactory therapeutics for the prevention or treatment of LF [16]. Recently, this hitherto neglected tropical disease has received significant attention and funding for research into the development of medical countermeasures (MCM). The recent attention and funding is partly due to its selection as a priority pathogen for accelerated vaccine development by the WHO R&D blueprint for action to prevent epidemics. Organizations such as the Coalition for Epidemic Preparedness Innovation (CEPI) have recently issued calls for proposals for the development of candidate vaccines against LASV [17]. CEPI aims to fund LASV vaccine research activities from preclinical proof of concept (POC) through phase II clinical trials. CEPI has also committed to the procurement of vaccine candidates and their warehousing in a “stockpile” that could ultimately be deployed in epidemics to reduce illness and death in humans. The more advanced of LASV vaccine candidates have already commenced first in human (FIH) studies in the USA and Belgium [18, 19], while other candidates are poised to enter clinical trials.

With the emergence of LASV vaccine candidates [16, 20], and the renewed desire to ensure clinical trials take place in affected countries, R&D investments in Africa are expected to witness a boost. Hence, this feasibility study was carried out to assess the presence and level of preparedness of clinical research sites in affected countries to conduct LF vaccine clinical trials. Currently, identification of ideal clinical trial sites for a proposed trial involves a convenient, contract research organization (CRO)-facilitated mapping of potential research sites and an assessment using a questionnaire [21]. This process often utilizes a “go/no go” approach that sees “unqualified” sites ruled out without priority given to gaps and capacity building needs of these facilities. It may also be open to bias with CROs likely to approach traditional sites with which they have some familiarity. This study, while utilizing a similar approach to the CROs, differs in the following ways:

- The use of relevant disease consortia/networks and social media to reach a wider range of potential research sites.
- Utilization of an innovative scoring template with emphasis on sites’ capabilities directly related to the conduct of LASV vaccine trials.
- Categorization of sites based on their strengths as related to LF clinical research such that many sites that are traditionally strong in LF research and with interest in conducting LF vaccine trials, but without significant track record of clinical trials were not excluded.
- Activity generated data which could be further analyzed to highlight capacity gaps of identified research sites with a view to implementing capacity building activities.

Europe and North America are likely to continue to host first in human (FIH) studies of vaccine candidates due to the scientific and regulatory rigor involved in ensuring initial safety, and proof of concept immunogenicity data are generated before proceeding to vulnerable populations [22].

Recent experience with LF disease epidemiology suggests that a phase III efficacy trial is a likely requirement for licensure [23]. As a result, the assessment of these sites is premised on their ability to conduct phase II and III trials. The WHO/TDR competency wheel was developed to provide a flexible framework of competencies that are needed to be demonstrated by a research site or team to carry out a successful clinical study. This study is an attempt to operationalize this framework. This study, while not being hypothesis driven, represents the first comprehensive attempt to systematically and operationally characterize clinical trial sites in LF-affected countries in West Africa.

The fact that only 44 sites were mapped in 8 countries indicates limited clinical trial capacity in LF-affected countries (Table 2). “A” represent site adjudged as ready to conduct LF vaccine trials. All the mapped sites in Burkina Faso (*n* = 5) and Mali (*n* = 2) were in this category. It also included 4 of the 5 sites characterized in Ghana. The scenario was different for Nigeria, where only 3 of 14 sites could be assigned to this category, and Liberia and Sierra Leone with no site in this category. Another common theme to most of the sites in these 3 countries was the lack of history of significant vaccine clinical trials. While Mali had conducted almost 100 clinical trials in its 2 sites over the past 30 years, sites in Nigeria, Liberia, and Sierra Leone—the 3 worst-hit LF countries—had cumulatively conducted only 56 clinical trials within the same period with only 6 of these being vaccine trials, as against 68 in Mali and Burkina Faso. Category A sites had well-developed processes, facilities, and qualified scientific and support staff. The National Institute of Health, Bill and Melinda Gates Foundation, Centre for Disease Control, US Agency for International Development, European and Developing Countries Clinical Trial Partnership (EDCTP), and the Partnership for Transforming Health (PATH) were the major global health institutions supporting clinical trials in the A category sites, while GlaxoSmithKline (GSK) and Novartis were the main private sector sponsors. It was instructive that 9 sites, including 2 category A sites, had no history of private sector-sponsored vaccine or drug clinical trials. The 3 Nigerian sites in this category did not have a strong track record of conducting drug or vaccine clinical trials but had well-developed human and material resources and were hence able and willing to conduct phase II and III LASV vaccine clinical trials. This underscores the importance of our approach of site mapping and assessment compared to the traditional method. Category B sites were essentially A sites but lacked adequate facilities for advanced clinical and laboratory evaluation of study subjects or investigational product (IP) handling. For example, 4 sites in this category were incapable of conducting PCR test, 4 had no guaranteed access to intensive care, and 3 had no access to − 80 °C freezer for storing biological samples, while 10 sites had less than 3 of such refrigerators. While all the B sites (except 2) had experience conducting clinical trials, most were focused on academic research involving already licensed drugs or interventions, and not drug or vaccine development. However, they had qualified personnel but required capacity building and infrastructural upgrades. Due to the expected financial implication of upgrading these sites, partnership with relevant global health organizations and private pharmaceutical developers would be useful. “C” sites required significant upgrade in human resources, clinical operations, and infrastructure. No site in this category got the maximal score for scientific staff. They also had little to no experience conducting clinical trials.

**Table 2:** List of mapped sites

S/N	Center	Location
1	Centre de Recherche en Santé de Nouna (CRSN), Burkina Faso	Burkina Faso
2	Clinical Research Unit, Nanoro	Burkina Faso
3	Groupe de Recherche Action en Santé (GRAS)	Burkina Faso
4	Centre National de Recherche et de Formation sur le Paludisme (CNRFP)	Burkina Faso
5	Centre Muraz	Burkina Faso
6	Center for Vaccine Development	Mali
7	Malaria Research and Treatment Centre	Mali
8	Kintampo Health Research Center	Ghana
9	Navrongo Health Research Center	Ghana
10	Komfo Anokye Teaching Hospital	Ghana
11	Kumasi Centre for Collaborative Research, Kwame Nkrumah University of Science and Technology	Ghana
12	Jos University Teaching Hospital	Nigeria
13	Nigerian Institute of Medical Research, Lagos	Nigeria
14	Irrua Specialist Teaching Hospital	Nigeria
15	Kenema Government Hospital	Sierra Leone
16	34 Military Hospital, Wilberforce	Sierra Leone
17	Connaught Hospital, College of Medicine and Health Sciences	Sierra Leone
18	Institute of Human Virology of Nigeria	Nigeria
19	Consortium for Health Research and Advanced Training (CHERAT), Enugu	Nigeria
20	Institute of Advanced Medical Research and Training, University of Ibadan	Nigeria
21	Lagos University Teaching Hospital	Nigeria
22	Center for Clinical Care and Research/Walter Reed Program-Nigeria	Nigeria
23	Federal Teaching Hospital, Abakaliki	Nigeria
24	Nigeria Global Health Trials Network, National Hospital Abuja	Nigeria
25	Center for Clinical Care and Clinical Research, Abuja	Nigeria
26	Laboratoire des fièvres hémorragiques	Guinea
27	Centre de Recherche et de Formation en Infectiologie	Guinea
28	Dodowa Health Research Centre	Ghana
29	Fondation pour la Recherche Scientifique (FORS)	Benin
30	Clinical Research Institute of Benin (IRCB)	Benin
31	The Liberia-US Clinical Research Partnership	Liberia
32	Phebe Hospital	Liberia
33	Center for Infectious Diseases Research, Bayero University, Kano	Nigeria
34	Noguchi Medical Research Center	Ghana
35	Centre Hospitalier Regionale Specialize (CHRS), Macenta	Guinea
36	University of Health and Allied Sciences	Ghana
37	Malaria Research Center	Ghana
38	Centre de recherche en épidémiologie, microbiologie et de soins médicaux (CREMS) de Pastoria à Kindia	Guinea
39	ELWA Medical Center	Liberia
40	Jackson F. Doe Hospital, Tapeta, Nimba County	Liberia
41	Federal Medical Center, Owo	Nigeria
42	Institute of Tropical Diseases Research and Prevention, University of Calabar Teaching Hospital	Nigeria
43	Hemorrhagic Fevers Laboratory in Guinea at Donka Hospital in Conakry	Guinea
44	MSF Epicenter Conakry	Guinea

Overall, most of the identified capacity gaps were in infrastructure, especially laboratory capacity to diagnose LF and funding. Steady power supply continues to be a challenge in West Africa, but all sites had backup plans to ensure uninterrupted power supply. All sites had access to laboratory facilities where routine hematological and parasitological parameters are carried out; 28 (80%) of the sites could perform PCR. Although many of the sites had a form of institutional ethics board, conduct of vaccine clinical trials usually requires national ethics committee and regulatory authority (NRA) approval.

The findings imply that strategic capacity strengthening measures are needed to enable the conduct of clinical trials and hence vaccine development in these countries.

### Limitations of the study

Although all attempts were made to ensure a high-quality non-biased study, completeness of the study was limited by factors such as slow or lack of response from the sites, reduced interactions due to the online nature of data collection, and the empirical nature of the scoring scale which was developed by the lead investigator based on his experience and prioritization of what was needed to conduct a successful Lassa fever vaccine trial. Also, the scorecard is yet to be validated; hence, we cannot be definite that a high score means that a site will be successful. Finally, some of the sites interpreted the question on history of clinical trials to basically refer to any study that utilizes a randomized clinical trial method irrespective of whether they were meant to evaluate an investigational product or not.

## Conclusion

A vaccine is urgently needed to prevent LF disease outbreaks, and its development should be a priority. While developing vaccine candidates requires significant financial investment in R&D, testing the vaccines in human populations requires the presence of research sites with human resources, facilities, and processes capable of conducting high-quality clinical trials. The results of this site survey indicated that most of the clinical trial sites were willing to perform vaccine trials in the future, provided capacity gaps can be addressed. Most of the identified gaps were within the purview of clinical and laboratory infrastructures, experience with sponsored vaccines and drug clinical trials, and investigational product handling. Partnership with pharmaceutical companies and relevant non-profit sector players is necessary to address these gaps. This experience with a systematic and comparative method of site assessment is encouraging, and the site selection method used can serve as guidance with points to consider for sponsors and researchers planning clinical trials. Site visits are ongoing to confirm the results of this survey. Until this process is complete, it may be difficult to conclude that the scorecard successfully predict which site could successfully hold vaccine trials. Further studies and publications are required to compare the data collected online with those obtained from actual site visits as well as to improve on the context of the questionnaire tool to improve its ability to gather more in-depth information as specified in the WHO/TDR competency wheel for clinical trials.

## Supplementary information

**Additional file 1:** Table S1 Scorecard template.

## Data Availability

The datasets used and analyzed during the study were archived at CEPI and are available from CEPI on reasonable request.

## Abbreviations

AVAREF: African Vaccines Regulatory Forum
CEPI: Coalition for Epidemic Preparedness Innovations
EDCTP: European and Developing Countries Clinical Trial Partnership
GCP: Good Clinical Practise
GSK: Glaxo Smithkline
ICTRP: International Clinical Trial Registry Platform
IP: Intellectual Property
LASV: Lassa virus
LF: Lassa fever
MCM: Medical countermeasures
MOU: Memorandum of Understanding
NIH: National Institute of Health
PCR: Polymerase Chain Reaction
R&D: Research and Development
RSA: Republic of South Africa
TDR: Tropical Disease Research
USA: United States of America
WHO: World Health Organization
