# Supplementary material for: A systematic scorecard-based approach to site assessment in preparation for Lassa fever vaccine clinical trials in affected countries
Source: Pilot Feasibility Stud. 2020 Feb 13;6:24. doi: 10.1186/s40814-020-00567-4 (PMC7020360; doi:10.1186/s40814-020-00567-4)
Supplement: Supplementary file 1 — Additional file 1: Table S1 Scorecard template. [file 40814_2020_567_MOESM1_ESM.docx]

Table 1: Score card template

| **CLINICAL TRIAL ORGANIZATION/SITE PROFILE** | | | | | | | **SCORE CARD** | **MAXIMUM POSSIBLE SCORE** | **JUSTIFICATION** |
| --- | --- | --- | --- | --- | --- | --- | --- | --- | --- |
|  | | | | | | |  |  |  |
| ***1. Site Overview*** | | | | | | |  |  |  |
| Name of site |  | | | | | |  |  |  |
| Location of main site |  | | | | | |  |  |  |
| Additional locations (satellite sites) | 1 | | | | | |  |  |  |
|  | 2 | | | | | |  |  |  |
|  | 3 | | | | | |  |  |  |
| Name(s) of Director(s) of the institution |  | | | | | |  |  |  |
|  |  | | | | | |  |  |  |
| Site Physical Address |  | | | | | |  |  |  |
| Site Postal Address |  | | | | | |  |  |  |
| Phone Number(s) |  | | | | | |  |  |  |
| Fax number(s) |  | | | | | |  |  |  |
| Website |  | | | | | |  |  |  |
| Name of contact person |  | | E-mail |  | | |  |  |  |
| Title/role of contact person |  | | Phone |  | | |  |  |  |
| Title/role of alternative contact person |  | | E-mail |  | | |  |  |  |
|  |  | | Phone |  | | |  |  |  |
|  | | | | | | |  |  |  |
| Indicate as appropriate: Yes / NO / N/A (not applicable) | | | | **Yes** | **No** | **N/A** |  |  |  |
|  | | | |  |  |  |  |  |  |
| Type of site | Hospital | Private | |  |  |  |  |  |  |
|  |  | Provincial | |  |  |  |  |  |  |
|  |  | Referral Teaching | |  |  |  |  |  |  |
|  |  | Military | |  |  |  |  |  |  |
|  | Laboratory | Private | |  |  |  |  |  |  |
|  |  | Government | |  |  |  |  |  |  |
|  | Clinical Research Centre | | |  |  |  |  |  |  |
|  | Other (specify) |  | | | | |  |  |  |
| ***2. Site accessibility*** | | | | **YES** | **NO** | **N/A** |  |  |  |
| Distance from airport (domestic/international) in km | | | |  | | | less 100km= 2, 100-200km=1, >200km=0 | 2 | Maximum score indicate that the site is close to an airport and easily accessible by a tarred road |
| Is the site accessible by road? | | | |  |  |  | Y= 1, N= 0 | 1 |  |
| What is the road condition (e.g. dirt, tarmac?) | | | |  | | | Tarmac=1, dirt= 0 | 1 |  |
|  | | | |  |  |  |  | **4** |  |
| *3.* ***Clinical research experience*** | | | | | | |  |  |  |
| How many years has this site been in existence? | | | |  |  |  | >= 10 = 4, 5-9= 3, 3-4= 2, 1= 1 | 4 |  |
| How many clinical trials has the site conducted? | | | |  |  |  | >= 10 = 4, 5-9= 3, 2- 4= 2, 1= 1 | 4 |  |
| How many are vaccine trials? | | | |  | | | >= 4 = 4, 3=3,2= 2, 1=1 | 4 | Premised on the assumption that for the conduct of Lassa fever vaccines clinical trial, site’s experience with vaccine trials is twice as important as drug trial experience |
| How many are drug trials? | | | |  | | | >= 8 = 4, 6-7= 3, 4-5=2, 1-3=1 | 4 |  |
| How many clinical trials does your site conduct at any one time | | | |  | | | 1-2= 2, 3-4= 1, >=5= 0 | 2 |  |
| List 5 more recent publications emanating from research conducted at the site | | | |  |  |  | >= 5 = 5, 4= 4, 3= 3, 2=2,1=1 | 5 |  |
| List top 5 pharmaceutical companies or funding agencies you have worked with | | | |  |  |  | >= 5 = 5, 4= 4, 3= 3, 2=2,1=1 | 5 |  |
|  | | | | | | |  | **28** |  |
| ***4. Staffing*** | | | | | | |  |  |  |
| What is the total number of scientific staff at the clinical research center? | | | |  | | | Divide Total number of staff by factor of 2 | 16 | Total number of scientific staff divided by a constant factor of 2 up to a maximum of 32 staff |
| What is the total number of support staff (including admin) at the clinical research center? | | | |  | | | Divide Total number of staff by factor of 2 | 16 | Total number of support staff divided by a constant factor of 2 up to a maximum of 32 staff |
|  | | | |  |  |  |  | **32** |  |
|  | | | | | | |  |  |  |
| ***6. Facilities*** | | | | | | |  |  |  |
| **6.1. Does your site have the following site facilities?** | | | | **Yes** | **No** | **N/A** |  |  |  |
| Administrative offices | | | |  |  |  | Y=1, N=0 | 1 |  |
| Access to reliable internet connection? State type (wifi/DSL/dongle/cable/satellite) | | | |  |  |  | Y=1, N=0 | 1 |  |
| Designated study document storage area and archiving facilities available on site (e.g. archiving room) | | | |  |  |  | Y=1, N=0 | 1 |  |
| Is there an established Quality Management System in place? | | | |  |  |  | Y=1, N=0 | 1 |  |
| Power source with dedicated functional back up available? | | | |  |  |  | Y=1, N=0 | 1 |  |
|  | | | |  |  |  |  | **5** |  |
|  | | | | | | |  |  |  |
| ***6.2. Clinical facilities*** | | | | | | |  |  |  |
| Outpatient care facility | | | |  |  |  | Y=1, N=0 | 1 |  |
| If yes, how many patients are generally seen during one day at the study/trial site? | | | |  | | |  |  |  |
| Inpatient care facility for study patients | | | |  |  |  | Y=1, N=0 | 1 |  |
| If yes, what is the bed capacity available to study patients? | | | |  | | |  |  |  |
| Dedicated work area for clinical research team | | | |  |  |  | Y=1, N=0 | 1 |  |
| Consenting or counselling room(s) | | | |  |  |  | Y=1, N=0 | 1 |  |
| Examination room(s) for subject evaluation and treatment | | | |  |  |  | Y=1, N=0 | 1 |  |
| Presence of or easy access to Intensive care unit? | | | |  | | | Y=1, N=0 | 1 |  |
| How many kilometers away is the ICU (if not on site)? | | | |  |  |  | Within site/less than 100km=2, >100km= 1, No access=0 | 1 |  |
| Emergency services (e.g where anaphylaxis can be handled)? | | | |  |  |  | Y=3, N=0 | 1 |  |
|  | | | |  |  |  |  | **8** |  |
| ***6.3. Investigational product handling*** | | | | | | |  |  |  |
| Vaccines Management (reception, local shipment), cold chain management, destruction, vaccination process and equipment. | | | |  |  |  | Y=1, N=0 | 1 |  |
| Dedicated +2 and +8-degree refrigerators? If yes indicate how many in spaces below. | | | |  |  |  | Y=1, N=0 | 1 |  |
| Access to minus 20 degrees Celsius freezers? If yes indicate how many in space below. | | | |  |  |  | Y=1, N=0 | 1 |  |
| Access to minus 70 degrees Celsius freezers? If yes indicate how many in space below. | | | |  |  |  | Y=1, N=0 | 1 |  |
|  | | | |  |  |  |  | **4** |  |
| ***6.4. Laboratory facilities*** | | | | | | |  |  |  |
| Access to Laboratory services? | | | |  |  |  | Y=1, N=0 | 1 |  |
| If no laboratories on site, what is the distance between the study/trial site and the laboratory(ies)? (Please indicate miles or kilometers.) | | | |  |  |  | <100km=2, >100km= 1 | 2 |  |
| List below the different laboratories available such as hematology, clinical  chemistry etc. | | | |  |  |  | Highest= 6, Lowest=1 |  |  |
| 1 Microbiology | | | |  |  |  |  |  |  |
| 2 Parasite Count | | | |  |  |  |  |  |  |
| 3 Hematology | | | |  |  |  |  |  |  |
| 4 Chemistry | | | |  |  |  |  |  |  |
| 5 Point of Care | | | |  |  |  |  |  |  |
| 6 Validation | | | |  |  |  |  | 6 |  |
| Is the laboratory able to conduct immunochemical studies e.g. RT-PCR? | | | |  |  |  | Y=1, N=0 | 10 | Yes=1 *10 (Ascribed a mark of 10 due to its importance in Lassa fever diagnosis/research |
| Does the laboratory(ies) have local/national reference ranges? | | | |  |  |  | Y=1, N=0 | 1 |  |
| Are you interested in conducting Lassa fever vaccine clinical trial in your center? | | | |  |  |  | Y=1, N=0 | 1 |  |
| Are you willing to provide further information about your organization/site by phone or email? | | | |  |  |  | Y=1, N=0 | 1 |  |
| CEPI is committed to protecting your personal data. Do you consent that the outcome of this survey be shared with funding organizations? | | | |  |  |  |  | **19** |  |
| Do you consent that the outcome of this survey be shared with other interested stakeholders? | | | |  |  |  |  | **100** |  |
